# Size rather than complexity of sexual ornaments prolongs male metamorphosis and explains sexual size dimorphism in sepsid flies

**DOI:** 10.1098/rspb.2022.2531

**Published:** 2023-05-10

**Authors:** Gowri Rajaratnam, Gerald Lui, Kathy F. Y. Su, Martin S. J. Chew, Yuchen Ang, Nalini Puniamoorthy, Patrick T. Rohner, Wolf U. Blanckenhorn, Rudolf Meier

**Affiliations:** ^1^ Department of Biological Sciences, National University of Singapore, Singapore; ^2^ Department of Evolutionary Biology & Environmental Studies, University of Zürich-Irchel, Winterthurerstrasse 190, 8057 Zürich, Switzerland; ^3^ Department of Biology, Indiana University, Bloomington, IN, USA; ^4^ Center for Integrative Biodiversity Discovery, Leibniz Institute for Evolution and Biodiversity Science, Museum für Naturkunde, Humboldt University, 10115 Berlin, Germany

**Keywords:** metamorphosis, sexual ornaments, development time, sexual dimorphism, growth, secondary sexual trait complexity

## Abstract

Male sexual ornaments often evolve rapidly and are thought to be costly, thus contributing to sexual size dimorphism. However, little is known about their developmental costs, and even less about costs associated with structural complexity. Here, we quantified the size and complexity of three morphologically elaborate sexually dimorphic male ornaments that starkly differ across sepsid fly species (Diptera: Sepsidae): (i) male forelegs range from being unmodified, like in most females, to being adorned with spines and large cuticular protrusions; (ii) the fourth abdominal sternites are either unmodified or are converted into complex *de novo* appendages; and (iii) male genital claspers range from small and simple to large and complex (e.g. bifurcated). We tracked the development of 18 sepsid species from egg to adult to determine larval feeding and pupal metamorphosis times of both sexes. We then statistically explored whether pupal and adult body size, ornament size and/or ornament complexity are correlated with sex-specific development times. Larval growth and foraging periods of male and female larvae did not differ, but the time spent in the pupal stage was *ca* 5% longer for sepsid males despite emerging 9% smaller than females on average. Surprisingly, we found no evidence that sexual trait complexity prolongs pupal development beyond some effects of trait size. Evolving more complex traits thus does not incur developmental costs at least in this system.

## 1. Introduction

Animals exhibit an impressive diversity of sexually dimorphic and morphologically complex traits whose rapid elaboration is thought to be responsible for the high species diversity of many clades [1,2]. Sexually dimorphic traits are widely employed for mate choice and often evolve rapidly, typically by sexual selection [3–6]. Sexual ornaments range from being purely ornamental structures to being functional weapons that contribute directly to individual reproductive success while presumably imposing fitness costs [7–9]. In arthropods there is substantial evidence that sexual traits are costly (e.g. armaments: [10]; courtship: [11]; contests: [12]; male signals: [13]; ejaculates: [14]). However, most studies focus on the cost of ornaments for adults, while fewer studies look at the developmental costs of building large and complex sexual ornaments (e.g. [10]). Yet, in a resource-limited world only a finite amount of resources is available for the development of the adult body, and any existing energy budget must be allocated to and traded off among various fitness-enhancing traits, thus incurring measurable life-history costs [15]. For instance, the sexually dimorphic abdominal grasping structures of male water striders (*Gerris odontogaster*) interfere with moulting so that males with longer processes take longer to emerge [16]. By contrast, a study of Japanese horned beetles *Trypoxylus dichotomus septentrionalis* found that male horns incurred no apparent costs in terms of developmental time or body size [17]. An earlier comparative study of sexual size dimorphism revealed that in some vertebrate groups (primates, birds) and flies, but not other insect groups (beetles, bugs, butterflies), males require more time than females to reach sexual maturity ([6,18]; see also [19]). While in vertebrates this can be explained by the larger body size of sexually reproductive males, male insects are typically (but not always) smaller than females [2]. The longer male development, especially of flies, was hypothesized to relate to greater developmental costs of producing male gonads, which at least in *Drosophila melanogaster* are larger and start developing earlier than female gonads [20], or the necessity of female income breeders to reach adulthood faster than males so as to start foraging for egg production sooner [6,21]. However, other developmental costs or trade-offs are conceivable, for instance that the complexity, rather than only the size of primary or secondary male sexual traits delays metamorphosis, which is investigated here.

Determining the costs of sexual ornaments is generally difficult in organisms that grow and feed continuously because resource allocation trade-offs can manifest in various, potentially unpredictable ways and traits [15,22]. This problem, however, is alleviated in holometabolous insects such as flies, beetles, or butterflies, where all adult structures are built in a closed energetic system during metamorphosis [10,23]. As the organism uses a finite amount of energy, which is obtained during their larval (foraging) period and subsequently reallocated within a defined pupal period for building all cuticular structures necessary for emerging as a complete adult, resource allocation can be interrogated by measuring development time, body size, ornament size and complexity.

**Figure 1.**
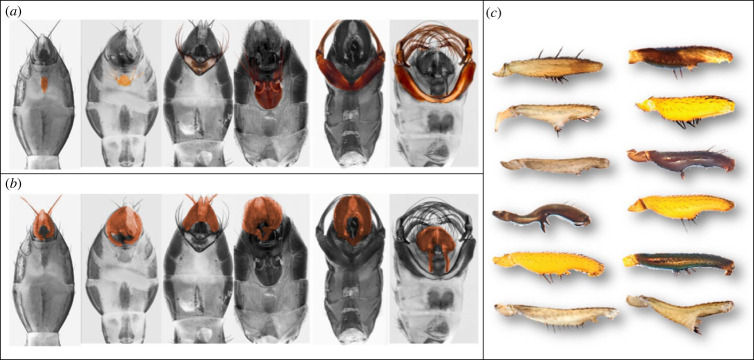
Males of selected sepsid fly species with variable sexual ornaments: ventral view of the abdomen showing (*a*) increasingly complex fourth sternite brush diversity from left to right and (*b*) corresponding genital clasper diversity (both highlighted in brown colour); and (*c*) diverse modified femurs of the male forelegs of various sepsid fly species with spines and undulations. Female forelegs are rarely ornamented (see most females on https://sepsidnet.biodiversity.online/ [24]. An exception is the females of *Themira lohmanus* with two prominent bristles on the fore femur).

Here we study a morphologically diverse family of black scavenger flies (Diptera: Sepsidae) with three sexually dimorphic ornaments that are built during the pupal stage, vary from simple to very complex, and are used by males during mating (figure 1). The first trait is the male foreleg. Given that mating is often costly, female sepsids are usually reluctant partners and employ various species-specific behavioural strategies of mate rejection and choice [25–35]. Thus, the armoured forelegs of many male sepsids serve to clasp the female wing base to aid males stay in position while the females, or possibly other males, might attempt to dislodge them [30,35–37]. The second trait are the fourth sternites, which can be unmodified [24,38] or converted into complex male structures that bear brush-like extensions resembling appendages (e.g. *Perochaeta dikowi, Nemopoda nitidula, Themira superba*). They are used to stimulate females before or during copulation [3,4,27–29,34,35]. Lastly, male sepsids have genital claspers that vary greatly in size, elaboration and precise stimulatory function [34,35,38]. All these sexual ornaments vary in shape, size and function across species, presumably to guarantee precise fit with a female of any size and successful copulations within species only (the lock-and-key hypothesis of [3,5,27,32,34,39]; cf. [1]; figure 1).

We carried out an extensive comparative analysis of 18 highly diverse species of sepsid flies (Diptera: Sepsidae; table 1; figure 1) to investigate whether the size and/or the structural complexity of these various male ornaments incurs developmental delays during their larval and/or pupal stage, thus ultimately proximately mediating the sexual size dimorphism of sepsid flies [2,6]. For instance, Rohner *et al*. [18] found that in less ornate species like *Sepsis fulgens* or North American *Sepsis punctum,* males are on average 3% smaller than females, while in more ornate species like *Themira superba* males are on average 13% smaller than the females. We evaluated morphological complexity using a novel type of Fourier analysis that quantifies how well Fourier representations with different harmonics capture trait shape (cf. [41–43]). Our results suggest that trait size often matters, but that size-independent trait complexity does not impose lasting developmental costs.

**Table 1.** Species used in this study (full names with their acronyms used), including provenance of the population, sample sizes for development times (and trait measurements), and oviposition periods applied (core dataset plus pilot dataset^b^).

species	locality	no. individuals	oviposition period (h)
*Allosepsis indica* AIND	Bali	103 (15)	2
*Archisepsis discolor* ADIS	Brazil	71 (5)	6
*Archisepsis pusio* APUS	Brazil	60 (5)	6
*Decachaetophora aenipes* DECA	USA	114 (15)	4
*Dicranosepsis sp.* DIC	Melaka	78 (15)	2
*Meroplius sauteri*^a^ MSAUT	Singapore	244 (14)	4
*Microsepsis armillata* MARM	Brazil	72 (20)	2
*Microsepsis mitis* MMIT	Costa Rica	50 (5)	6
*Nemopoda nitidula* NNIT	Hamilton, USA	96 (6)	6
*Perochaeta dikowi* PDIK	Pahang, Malaysia	33 (11)	2
*Sepsis cynipsea* SCYN	Sweden	82 (15)	2
*Sepsis fulgens* SFUL	Switzerland	48 (10)	6
*Sepsis neocynipsea* SNEO	USA	114 (15)	2
*Sepsis punctum* SPUN	Georgia, USA	56 (10)	6
*Themira biloba* TBIL	Germany	54 (5)	6
*Themira lucida* TLUC	Germany	128 (5)	6
*Themira minor* TMIN	Germany	91 (15)	2
*Themira superba* TSUP	Germany	50 (5)	6
*Meroplius fukuharai*^b^		67 (5)	—
*Parapaleosepsis plebeia*^b^		373 (5)	—
*Sepsis (Australosepsis) frontalis*^b^		58 (5)	—
*Sepsis (Australosepsis) niveipennis*^b^		86 (5)	—
*Sepsis dissimilis*^b^		540 (5)	—
*Sepsis monostigmata*^b^		82 (5)	—
*Sepsis thoracica*^b^		112 (5)	—
*Themira putris*^b^		108 (5)	—

^a^dropped from the phylogenetic generalized least squares analysis because not in Lei *et al*.'s [40] phylogeny.

^b^pilot dataset (some information missing).

## 2. Material and methods

All measured individuals were derived from long-term laboratory cultures of the various species derived from multiple live females originally collected in the regions specified in table 1. Methods for the general husbandry and species-specific rearing of sepsid flies have been reported in Puniamoorthy *et al*. [34,35] and Rohner *et al*. [18].

### (a) Harvesting embryos

A large Petri-dish of fresh dung was provided repeatedly to laboratory cultures containing groups of many flies of each species in large plastic containers. Females only had 2–6 h for oviposition (table 1), whereby species with lower egg output were flexibly offered longer oviposition windows. Several individual cohorts of multiple individuals of defined age of all species were thus obtained from eggs laid within this short time frame.

### (b) Larval period

Cultures were subsequently incubated on excess defrosted cow dung at a constant temperature of 25.0 ± 0.5°C and humidity of 21.5 ± 1 g m^−3^. They were left undisturbed for 3–7 days, depending on the (known) development time of each species, until 3rd instar larvae were observed. Twenty-four hours later, 40 to 60 pre-pupae were harvested daily for 3–4 days or until no additional pre-pupae were observed. Multiple harvests typically were necessary to precisely assess the larval period for both sexes.

An abdominal gas bubble released during pupal morphogenesis in cyclorrhaphan flies marks the precise start of the pupal phase: after its formation, pupation is no longer reversible [44], and the gas bubble is lost within 30 min. Pre-pupae were then harvested, cleaned in settled water with forceps, and aligned on a thin film of moist filter paper. Individuals were arbitrarily numbered to track their identity throughout the study (electronic supplementary material, figure S1). The corresponding time points of pupation and adult emergence were traced with time lapse cameras for each of the 1544 individuals in our study (table 1). We produced images of the pre-pupae at 10 min intervals with a Canon D80 camera mounted on a Nikon SMZ-1500 Stereoscopic Microscope with an LED bottom light until all pre-pupae had transited into the pupal stage. The start of pupal development was set when the gas bubble was no longer visible. This also defined the end point of the larval period. (The end of the bubble stage could not be determined for *Decachaetophora aeneipes* and *Themira minor* because their pupae are extremely sclerotized. For these two species the point of complete sclerotization was used as the time-point for entrance into pupation.)

We further present results of a previous pilot study testing additional species (last eight species in table 1), for which the methods differed slightly with regard to growth conditions (e.g. at 26°C). For this subset of data, only the sex-specific total development times and complexity of structures were determined, without separating larval feeding and pupal development time.

### (c) Pupal period

Rather than pupal mass, which is difficult to measure accurately without tight ambient moisture control, we used pupal volume as a proxy for the resources available for metamorphosis after termination of the larval foraging period (cf. [45]). Pupal size was measured at the start of pupation from the images taken using ImageJ ([46]; electronic supplementary material, figure S1), from which pupal volume was subsequently estimated using a formula for a perfect ellipsoid: 1/6π*(pupal width)^2^*(pupal length). All pupae remained in the incubator at 25°C until emergence (for 4–7 days, depending on species). Prior to expected adult emergence, pupae were removed and placed into individual glass vials with adequate moisture. Time-lapse images of these vials were then again taken at 10 min intervals to ascertain the precise time point of adult eclosion, which determined pupal duration (in hours) with an error margin of 20–30 min.

After emergence, flies were transferred into a large Petri-dish containing sugar water, where they were left for *ca* 24 h until fully sclerotized with an inflated abdomen, which facilitated the quantification of all cuticular structures. After having fed on sugar water, all flies were killed, sexed and preserved in 70% ethanol. The scutum width of all specimens was measured as an index of body size using the Leica MZ16A Microscope and Application Suite software (electronic supplementary material, figure S2)

### (d) Quantifying size and complexity of claspers, fore-femurs and 4th sternites

Larval cohorts for any species were considered sufficiently large only if more than 30 males and 30 females emerged as adults (with the exception of *Perochaeta dikowi,* which exhibited high mortality). Larval and pupal durations were measured for all available individuals, but the labour-intensive assessment of morphological complexity was only carried out for a subset of 5–20 (on average 10) adult males and females derived from several cohorts per species so as to well capture the intra-specific variance in development time and body size (sample sizes in table 1). These specimens were dissected (removal of fore-femurs, sternites and claspers for males; fore-femora and sternites for females) and imaged with a digital camera at high resolution. The images were then traced to produce detailed two-dimensional outlines of all above-named traits. Individual trait size was measured by its total pixel count from each image using Adobe Photoshop CS6.

**Figure 2.**
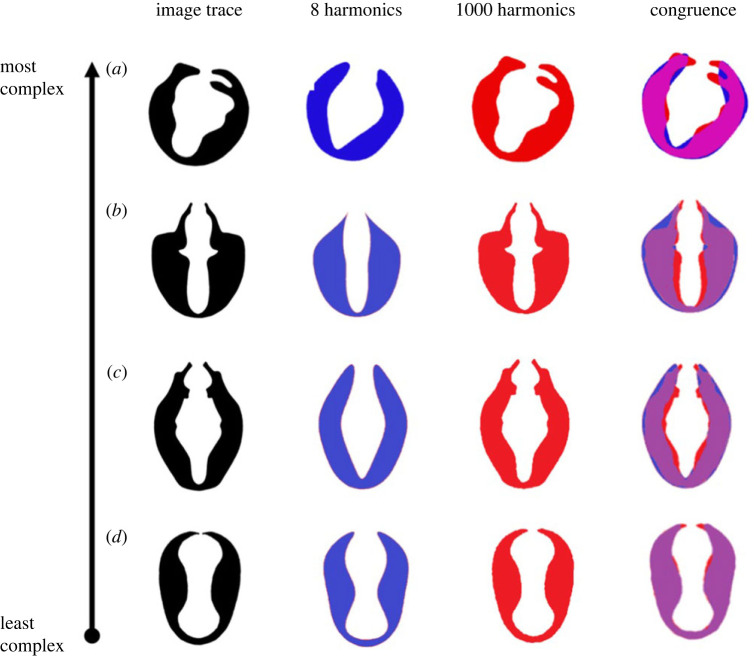
Comparison of Fourier approximations at two different harmonic levels (8 versus 1000) to quantify the (size-corrected) morphological complexity across a range of genital claspers. The ratio of non-congruent (blue versus red) to congruent (purple) areas was our score of complexity. (*a*) For *Microsepsis armillata*, with a complex genital shape, reconstructions at 8- and 1000-harmonics showed a large extent of disagreement, producing a high complexity score of 0.320; (*b*) for *Decachaetophora aeneipes* the complexity score was 0.285; (*c*) for *Meroplius sauteri* the complexity score was 0.252; and (*d*) for *Allosepsis indica*, with the least complex clasper shape, the disagreement between the 8- and 1000-harmonics reconstruction was very small, yielding a low complexity score of 0.129.

Trait shape complexity was estimated by comparing Fourier reconstructions with different (8 versus 1000) harmonics. The accuracy of a Fourier reconstruction depends on the number of harmonics. An 8-harmonics reconstruction of a complex shape like a clasper yields a poor approximation, whereas a reconstruction with 1000 harmonics yields a precise outline that closely matches the original trace (figure 2). For complex shapes, the degree of dissimilarity between the 8- and 1000-harmonics reconstructions is thus expected to be large, while these differences should be small(er) for simpler shapes. Shape complexity therefore can be quantified based on this ratio of non-congruent to congruent Fourier-estimated areas. Figure 2 exemplifies this process for genital claspers: the more complex clasper of *Microsepsis armillata* generates a higher complexity score (0.320) than the simple clasper shape of *Allosepsis indica* (0.129).

2The variable male sternite and fore-femur complexity scores (but not those of the claspers, which only occur in males) were normalized against the respective female structures as the baseline (male/female) for each species to account for sexual dimorphism. The only exception were *Perochaeta dikowi* females, which lack sclerotised fourth sternites and thus were given an arbitrary score of 1 for sternite complexity to be used for normalization.

### (e) Statistical analyses

To investigate putative developmental costs of the complexity of the three ornaments in terms of larval, pupal, or total development time, separate multiple phylogenetic generalized least squares (PGLS) regressions (as implemented in the R package *caper*; [47]) based on the phylogeny published by Lei *et al*. [40] were carried out using overall species averages. This controls for the shared evolutionary history of all species. (We had to drop *Meroplius sauteri* because this species is missing in that phylogeny.) The mean absolute (male) body size (i.e. scutum width) of the species, as well as the sex difference in body size (i.e. sexual size dimorphism), were both included as covariates in this analysis to control for known relationships of body size and dimorphism with sex differences in overall development time and/or growth rate (which were not the focus here: [6,18]). Alternatively or additionally, we controlled for trait size (which typically scales with body size) or trait size dimorphism instead of body size, or for both. All statistical analyses were conducted in R ([48]; all ANOVAs are presented in the electronic supplementary material, tables S1–S3).

## 3. Results

### (a) Sex differences in larval and pupal development, growth rate and body size

**Figure 3.**
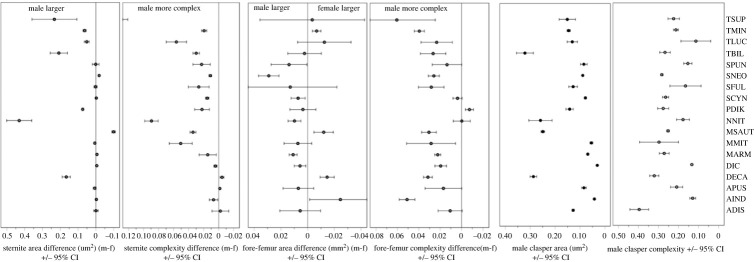
Sexual fourth sternite size and complexity difference (left), fore-femur area and complexity difference (middle), and male clasper area and complexity (right) for the 18 core sepsid species used in our study (see table 1 for species acronyms, figure 1 for traits).

The sepsid species assessed here revealed their typical female-biased sexual size dimorphism (SSD), based on both scutum width and pupal volume ([6,18]; electronic supplementary material, figure S3). Across all 18 species of the main dataset (table 1), the average difference between male and female scutum width (i.e. body size; electronic supplementary material, figure S2) was –0.134 ± 0.042 mm (±95% confidence interval (CI); one-sample *t*-test *p* < 0.001), and −0.216 ± 0.255 mm^3^ for pupal volume (*p* = 0.114). By contrast, the average sex difference in sternite area (0.62 ± 0.58 mm^2^; *p* = 0.052) was male-biased, while that for fore-femur area was essentially zero (0.002 ± 0.006 mm^2^; *p* = 0.452; figure 3). (Claspers only occur in males.) Regardless, the sternites (0.029 ± 0.016; *p* = 0.002), forelegs (0.024 ± 0.008; *p* < 0.001), and claspers (0.231 ± 0.035; *p* < 0.001) of males were always more complex than the female equivalent (no measurements for claspers; figure 3).

At the same time, the duration of the larval period (−0.55 ± 2.28 h; −0.26% mean difference; *p* > 0.2) did not differ between the sexes, whereas pupal duration reflecting metamorphosis (7.42 ± 4.50 h; 4.69%; *p* = 0.005), and consequently also total development time (=larval + pupal duration; 6.70 ± 5.38 h; 1.83%; *p* = 0.026), were significantly prolonged for males (electronic supplementary material, figure S4). Female-biased SSD in combination with longer development of males implies faster growth rates of females (0.000449 ± 0.000484 mm h^−1^; *p* = 0.087; −3.72%; cf. [6]).

### (b) Developmental costs of sexually dimorphic ornaments

Of all reared flies (electronic supplementary material, figure S1), we used 5 to 15 male and female individuals to measure the three adult traits (figure 1) for all the 18 differently ornamented sepsid fly species (to which we were able to add preliminary data for eight additional species stemming from a previous similar pilot study, as internal replication; table 1). In the end, individual data were averaged to obtain sex-specific species means for all traits for our comparative analysis across species. We further computed the mean sex differences (male minus female) for all traits of all species, which best characterizes the sexual dimorphism of male relative to female traits, as females may have modified traits as well in this species group (forelegs and sternites only; cf. figure 1). Using sex differences simplified our statistical analysis so as to focus on the evolution of the male traits *relative* to the same trait of the female. This even allowed analysing traits that are only expressed in males (i.e. the genital claspers), but for completeness we also analysed male traits without reference to females.

Trait size means (pupal volume, scutum width, sternite, clasper, and foreleg area) were generally positively correlated across all 18 species (*r* = 0.43−0.87; all *p* < 0.05), as expected from morphological scaling. In a first analysis of size effects only across species, male sternite size (area) correlated positively with both larval (*r* = 0.44; *p* = 0.089) and especially pupal (*r* = 0.62; *p* = 0.011) development time, regardless of whether we simultaneously controlled for body size (scutum width). This contributes to the overall longer development times of males versus females (*r* = 0.24; *p* = 0.003). Male foreleg area only prolonged larval (*r* = 0.58; *p* = 0.020) but not pupal or total development time (*p* > 0.3); whereas male genital clasper area prolonged larval (*r* = 0.68; *p* = 0.004) and marginally also pupal (*r* = 0.43; *p* = 0.090), but not total development time (*p* > 0.2).

**Figure 4.**
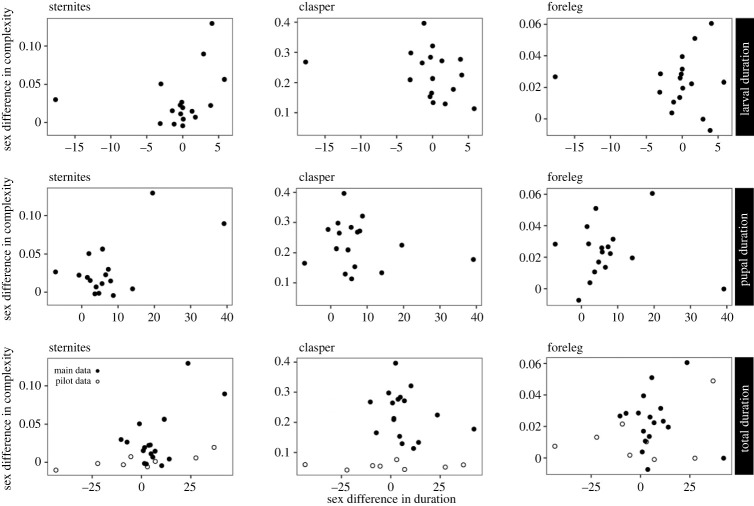
Relationship of the mean sexual difference (male-female) in structural shape complexity of three sexual traits (fourth sternite brushes (left), male genital claspers (middle), male fore-femur ornamentation (right); cf. figure 1) with the corresponding sexual difference in larval (top row), pupal (middle row), or the resulting overall development time (bottom row). The bottom plots for total development time include eight additional species from a pilot study (white circles) in addition to our core data for 18 (17) species (black circles; table 1). Only in some analyses sternite complexity was significantly correlated with pupal and total development time differences between the sexes, suggesting a developmental (i.e. metamorphosis) cost of male sexual ornament complexity independent of body or trait size (dimorphism).

Our final analysis focused on trait complexity while controlling for absolute body size (scutum width) and sexual size dimorphism across species. Only fourth sternite complexity difference between the sexes correlated positively with the sex differences in pupal duration, suggesting a developmental cost of ornament complexity independent of body size and dimorphism (supp. ANOVA electronic supplementary material, table S1; figure 4). There was no such correlation with the larval duration difference (electronic supplementary material, table S1; figure 4). Nonetheless, the effect of male sternite complexity on pupal duration was strong enough to significantly prolong the total development time difference between the sexes (i.e. the sum of larval and pupal development time), both when considering the core data set of 18 species (PGLS: 17) and when adding eight additional species from the earlier pilot study (table 1; electronic supplementary material, table S1; figure 4). When using sternite size instead of or in addition to body size in the analyses, the effect of sternite complexity on pupal duration disappeared, and a positive effect of sternite size appeared. This is in line with the aforementioned analysis based on body size only and suggests a developmental cost in terms of prolonged metamorphosis conceivably needed for building a larger male sternite plate (electronic supplementary material, table S1; sternite size and complexity are merely weakly positively correlated: *r* = 0.26). By contrast, clasper and fore-femur complexity did not show any across-species relationship with larval, pupal or total development time, whether body size (dimorphism) and/or trait size (dimorphism) were entered as covariates (electronic supplementary material, tables S2, S3). Thus, for these two sexual traits (ornaments) only trait size (i.e. foreleg or genital clasper area) showed a significant positive (i.e. prolonging) effect on the male larval (foraging) period, but not necessarily the pupal period, when analysing male traits only (electronic supplementary material, tables S2, S3; cf. above size analysis). These results were generally not strongly influenced by high pairwise correlations among the key explanatory variables (scutum size, trait size, trait complexity) when used simultaneously in the analyses presented in the electronic supplementary material tables S1–S3 (*r* = −0.27 to 0.26 for sternites, *r* = 0.17 to 0.65 for forelegs and *r* = −0.02 to 0.25 for claspers).

Finally, the majority of our statistical models indicated no phylogenetic signal (electronic supplementary material, tables S1–S3), suggesting that the considered sexual traits evolved largely independently of phylogenetic relatedness in this (sub)group of sepsid flies. We therefore show the raw data in all plots in figure 4. Overall we conclude that trait complexity of any of our studied (male) secondary sexual characters does not produce detectable developmental costs beyond some effects of body or trait size *per se* (electronic supplementary material, tables S1–S3; figure 4).

## 4. Discussion

The fitness benefits associated with large body size and sexual size dimorphisms have been studied in many taxa [2,6,19]. By contrast, comparatively little is known about the developmental origins of dimorphism. Does the smaller sex feed for a shorter time to develop faster, or is its smaller size mediated by other types of energetic costs or trade-offs during development? We here investigated this by monitoring the sex-specific development of larvae and pupae of 18 species of sepsid flies (table 1) to relate this information to the morphological complexity and size of their adult male secondary sexual traits (ornaments). We found the larval feeding period (i.e. larval development time) to be roughly the same for males and females of most species (mean difference: 0.26%; electronic supplementary material, figure S4). Nevertheless, adult males emerged on average *ca* 9% smaller and 5% later from the pupal stage, thus requiring a few additional hours for metamorphosis in the pupa (electronic supplementary material, figure S4). This confirms earlier studies on various taxa including sepsids [6,18,19,21]. Crucially however, and contrary to our original hypothesis and intuitive notions, this delay was largely unrelated to the structural complexity of any of the three sexual ornaments investigated (fourth abdominal sternites; modified forelegs; male genital claspers; figure 1), all of which are primarily or exclusively expressed in males. It thus appears that male sepsids in general do not pay heavily during their ontogeny for the construction of elaborate (sexual) ornaments, for instance by requiring more time and/or energy for metamorphosis during the pupal stage, as tested here (cf. [45]).

We here assessed sexual trait complexity with a novel kind of Fourier analysis (compare [41–43]). This was necessary because the morphological variability of sepsid sexual ornaments is so high that no homologous landmarks can be defined that would be valid across genera. Overall, our method using Fourier descriptors quantified structural trait complexity well in a way that matches qualitative expectations (see figures 1–3). However, it is important to remember that we are quantifying three-dimensional morphological structures based on two-dimensional outlines. We thus cannot entirely exclude that our largely negative findings of low to no developmental costs of secondary sexual trait complexity may be a consequence of our quantification method. However, it is also conceivable that complex sexual ornaments can be produced by fairly simple developmental pathways (cf. [10]). This would mean that the postulated relationship between the complexity of an ornament and developmental delays may not be valid. Indeed, this would be the major finding of our study if the Fourier method indeed appropriately quantified morphological complexity. Further research using this method in other contexts will be needed to evaluate the performance of our trait complexity estimates relative to traditional landmark morphometrics (if applicable), especially how it relates to the developmental processes that generate complex adult structures and their associated costs.

SSD is commonly observed in species with separate sexes, but the underlying developmental mechanisms creating it are poorly understood even though body size is well known to substantially affect reproductive success in most organisms [2,18,19,49]. Larger females typically lay more eggs [50], whereas smaller males often have reduced mating success [32,51,52] and/or are more vulnerable to predation or death from malnourishment [53]. The energetic costs of producing larger (internal) male (relative to female) gonads had previously been conjectured to contribute to the female-biased SSD of many invertebrates, but empirical evidence is sparse [6,20]. We here provided some evidence for further, externally visible primary and secondary sexual traits (forelegs, genital claspers, sternites) causing larval or pupal developmental delays in sepsid flies, although future studies should include internal sexual traits (such as gonads) because effects of one type of trait could ultimately statistically mask effects of another (e.g. here gonad investment masking sexual trait complexity) in affecting sex-specific developmental and growth rates. As at least in some species females are also ornamented (e.g. female *Themira putris* and *Themira lohmanus* on https://sepsidnet.biodiversity.online/), we here moreover made sure to assess the developmental costs of male secondary sexual trait complexity and size relative to females as the baseline, to ultimately better understand how sexual dimorphism may result from different developmental pathways of males and females with divergent morphologies ([2,6,18,19,21; e.g. [54–56]).

We had expected that the males of a majority of sepsid species emerge smaller because they feed for a shorter period as larvae and consequently pay a developmental cost for forming elaborate sexual ornaments during (pupal) metamorphosis. However, we found the larval feeding period (i.e. development time) not to differ between the sexes, contrary to the general pattern found for many insects [19]. Yet sepsid males tend to emerge smaller than their female conspecifics. This implies that sepsid males as a rule do not compensate for their smaller size by feeding longer as larvae, and that females apparently grow faster and/or convert food more efficiently into body size [6,18,21]. The main difference between the sexes documented here is that males extend their pupal period by *ca* 5%, nevertheless forming *ca* 9% smaller pupae and adults in the end (electronic supplementary material, figure S3), time that we originally expected to be required for the construction of their complex sexual ornaments (including large brushes, spines or cuticular undulations etc.; figures 1, 3, and 4). However, sexual trait shape complexity did not generally extend the pupal period or metamorphosis here, merely weakly only for the ornamented fourth sternites (figure 1). Thus, any developmental delay signifying a cost of male secondary sexual trait complexity overall appears to be minor in sepsid flies. This may be partially explained by recent findings that merely minor changes in the relative sizes of histoblast nests are required for the development of sternite brushes of very different size [57,58].

In conclusion, the duration of development is an important individual fitness component that is influenced by a multifaceted array of intertwined extrinsic and intrinsic factors to be integrated as animals develop ([6,19,20]; e.g. [54–56]). Faster life histories are generally advantageous in growing populations. Individuals that mature earlier have more time to find a mate (both sexes) and oviposit (females only). Especially if the mating season is brief and females are reluctant to mate multiply, insect males are often selected to emerge earlier (so-called protandry), typically at smaller size because the growth period is curtailed [21,59,60]. On the other hand, it takes time and is energetically costly to develop and grow to a larger size, and the longer an individual spends in the juvenile, i.e. larval or pupal stage, the greater the risk of predation, parasitism, or stochastic death [45]. Nevertheless, our comparative study of sepsid flies ultimately suggests that all these presumed (and sometimes demonstrated) mortality factors are not sufficient to produce significant developmental delays in response to amplified morphological secondary sexual trait shape complexity. Turned around, this argument implies that lacking developmental costs may explain why complex sexual ornaments are so variable in shape in nature. Our study indicates that the costs of producing elaborate secondary sexual traits are overall lower than costs of producing larger trait or body sizes.

## Data Availability

Data analysed for this paper ‘CostOfOrnamentComplexityDataSpeciesMeans' are found under https://doi.org/10.5061/dryad.h18931zrc [61]. The data are provided in the electronic supplementary material [62].
